# Complete genome sequence of *Afipia carboxidovorans* strain SH125, a non-denitrifying nitrous oxide-reducing bacterium isolated from anammox biomass

**DOI:** 10.1128/mra.01279-23

**Published:** 2024-02-22

**Authors:** Kohei Oba, Shohei Yasuda, Akihiko Terada

**Affiliations:** 1 Department of Applied Physics and Chemical Engineering, Tokyo University of Agriculture and Technology, Koganei, Tokyo, Japan; 2 Civil Engineering, School of Engineering, College of Science and Engineering, University of Galway, Galway, Ireland; 3 Global Innovation Research Institute, Tokyo University of Agriculture and Technology, Fuchu, Tokyo, Japan; University of Southern California, USA

**Keywords:** nitrous oxide, *nosZ*, anammox, denitrification, N_2_O-reducing bacteria

## Abstract

Here, we report a genome sequence of *Afipia carboxidovorans* strain SH125 isolated from an anammox reactor. This facultative anaerobic strain possesses the clade I-type nitrous oxide (N_2_O) reductase gene, devoid of nitrite- and nitric oxide reductase genes. Deciphering the genome will help explore N_2_O reducers instrumental in N_2_O mitigation.

## ANNOUNCEMENT

**Fig 1:**
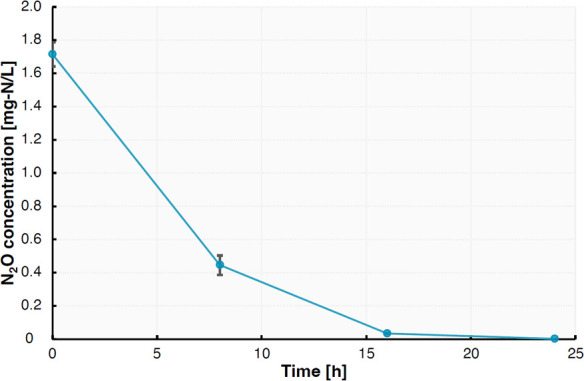
Time course of N_2_O concentration by *A. carboxidovorans* strain SH125 during batch culture under anaerobic conditions. Solid circles represent N_2_O concentrations. Each error bar represents a standard error of the mean. The experiment was conducted in triplicate.

N_2_O-reducing bacteria are the primary consumers of N_2_O (1), a highly potent greenhouse and ozone-depleting gas (2, 3). More descriptions regarding the phylogeny, functions, and physiologies of N_2_O-reducing bacteria toward mitigating N_2_O emissions are required (4, 5). *Afipia carboxidovorans* strain SH125, detected as a candidate for N_2_O sink in soils (6) and engineered systems (7, 8), was obtained from anammox biomass enriched by exogenous N_2_O supply (9). The biomass was serially diluted with 20× diluted phosphate-buffered saline and spread onto 1.0 wt% gellan gum plates containing the anammox medium (10). Cultures were anaerobically grown using a jar filled with a deoxygenating reagent (Anaeropack, Mitsubishi Gas Chemical, Tokyo, Japan) and N_2_O gas [5% (vol/vol)], followed by colony picking. The isolate showed N_2_O consumption activity when N_2_O was added to Japan Collection of Microorganisms (JCM) Medium No. 12 (nutrient broth medium with 0.5% NaCl, pH = 7.5) under an anoxic condition (Fig. 1). This activity test, referring to reference (11), was initiated at 30°C by adjusting the headspace gaseous N_2_O concentration of 1.7 mg N/L.

After an aerobic incubation using JCM Medium No. 12, the genome was extracted with a phenol-chloroform method (12, 13) and purified by a CTAB/NaCl solution. RNA as a contaminant in the genomic DNA was decomposed by RNaseA (TaKaRa Bio, Inc., Shiga, Japan). Barcoding and library preparation were conducted using Native Barcoding Expansion 1–12 EXP-NBD104 [Oxford Nanopore Technologies (ONT), Oxford, UK] with ONT Long Fragment Buffer and ONT Ligation Sequencing Kit SQK-LSK109. Sequencing was conducted on an R9.4.1 flow cell with the MinION Mk1B. Basecalling was performed using Guppy v6.5.7 (https://community.nanoporetech.com/downloads) with a super-accurate model (options –config dna_r9.4.1_450bps_sup.cfg -x cuda:0). Subsequently, demultiplex and barcode sequence removal was performed by applying Guppy’s guppy_barcoder command. This resulted in 71,104 raw reads (1,373,410,240 bp), with an *N*_50_ value of 41,150 bp. NanoFilt v2.8.0 (14) was used to filter low-quality reads (*Q* < 12) and short reads (<10,000 bp). Error correction was performed using Canu v2.2 (15), and genome assembly was generated by Flye v2.9.3 (16) (option –nano-corr). The assembly was further polished using Medaka v1.11.2 (https://github.com/nanoporetech/medaka) (option -m r941_min_sup_g507). Completeness (99.68%) and contamination (0.00%) were evaluated with CheckM v1.2.2 lineage_wf (17). DDBJ Fast Annotation and Submission Tool v1.6.0 (18, 19) was applied for annotation. Default parameters were used for all software unless otherwise specified.

The genome consisted of a single circularized contig with a length of 3,743,720 bp (284-fold coverage) and G + C content of 62.3%. The genome was predicted to encode 3,682 protein-coding sequences, 3 rRNA genes, and 51 tRNA genes. The genome annotation did not identify any nitrite reductase and nitric-oxide reductase but a clade I N_2_O reductase. The denitrifying genotype suggests the strain is a non-denitrifying N_2_O-reducing bacterium. The genome sequence of *A. carboxidovorans* strain SH125 will contribute to extending a comprehensive understanding of its role as an N_2_O sink.

## Data Availability

This genome sequence has been deposited on DDBJ under the accession number no. AP029055. Sequencing data are available in the Sequence Read Archive under accession number no. DRR517369.
